# Coupling Peptide Antigens to Virus-Like Particles or to Protein Carriers Influences the Th1/Th2 Polarity of the Resulting Immune Response

**DOI:** 10.3390/vaccines4020015

**Published:** 2016-05-05

**Authors:** Rattanaruji Pomwised, Uraiwan Intamaso, Martin Teintze, Mark Young, Seth H. Pincus

**Affiliations:** 1 Department of Microbiology, School of Medicine, Prince of Songkla University, Hadyai, Songkla 90110, Thailand; rattanaruji.p@psu.ac.th; 2 Faculty of Allied Health Sciences, Burapha University, Bangsaen, Chonburi 20131, Thailand; uraiwani@buu.ac.th; 3 Department of Chemistry and Biochemistry, Montana State University, Bozeman, MT 59717, USA; mteintze@montana.edu; 4 Department of Plant Sciences, Montana State University, Bozeman, MT 59717, USA; myoung@montana.edu; 5 Departments of Pediatrics and Microbiology, School of Medicine, Louisianna State University, New Orleans, LA 70118, USA

**Keywords:** group B streptococcus, *Streptococcus agalactiae*, capsular polysaccharide, antigen, mimotope, synthetic peptide

## Abstract

We have conjugated the S9 peptide, a mimic of the group B streptococcal type III capsular polysaccharide, to different carriers in an effort to elicit an optimal immune response. As carriers, we utilized the soluble protein keyhole limpet hemocyanin and virus-like particles (VLPs) from two plant viruses, Cowpea Chlorotic Mottle Virus and Cowpea Mosaic Virus. We have found that coupling the peptide to the soluble protein elicits a Th2 immune response, as evidenced by the production of the peptide-specific IgG1 antibody and IL-4/IL-10 production in response to antigen stimulation, whereas the peptide conjugated to VLPs elicited a Th1 response (IgG2a, IFN-γ). Because the VLPs used as carriers package RNA during the assembly process, we hypothesize that this effect may result from the presence of nucleic acid in the immunogen, which affects the Th1/Th2 polarity of the response.

## 1. Introduction

To elicit optimal antibody responses to small peptide immunogens, they are often attached to larger carriers, either by chemical conjugation or genetic fusion. We have previously identified a peptide, designated S9, that mimics the structure of a protective epitope of the group B-streptococcus (GBS) capsular polysaccharide type III [1,2]. In an effort to present this peptide in a conformation that might mirror that of the natural, repetitive epitope, we sought to express it on the surface of a virus consisting of repeating capsid protein subunits. For this purpose, we chose to use Cowpea Chlorotic Mottle Virus (CCMV), a member of the Bromoviridae virus family, whose three-dimensional (3D) structure has been solved [3]. The capsid is composed of 180 chemically identical protein subunits. Each protein subunit is comprised of an eight-stranded, antiparallel, β-barrel core (from amino acids 52–176), and results in the outward projection of five loops. The DNA encoding each loop has been engineered to have *Bam*H I restriction enzyme sites to allow for the insertion of DNA encoding epitopes. Salt and pH stable mutants of CCMV have been identified [4]. Both wild-type and mutant viruses can be expressed in heterologous expression systems [5]. A structurally similar virus, Cowpea Mosaic Virus (CPMV), has been proposed as a vaccine scaffold [6,7]. CPMV has a stable structure and possesses a single uniquely reactive lysine residue per asymmetric unit (60 per virus particle) which may be used for chemical conjugations [7,8,9]. In this study we have conjugated the S9 peptide to CCMV and CPMV virus-like particles (VLPs), to CCMV capsid monomers, and to the soluble carrier protein keyhole limpet hemocyanin (KLH). We then studied the immune response these antigenic preparations elicit in mice. We have found that in the absence of adjuvant, the carrier has an important effect upon the polarity of the anti-peptide response, with the VLPs eliciting a Th1 response, and KLH a Th2 response.

## 2. Materials and Methods

### 2.1. Virus and VLP Production

Infectious CCMV capsid protein chimeras, with peptides inserted into one of the exposed loops at AA positions 62, 102, 114, 129 or 169, were produced using plasmids encoding RNA3. Plasmids encoding CCMV RNA1, RNA2, and wild-type and chimeric RNA3s were linearized and transcribed *in vitro* using T7 Megascript (Ambion, Austin, TX, USA). Mixtures of RNA1, RNA2, and RNA3 were spotted on carbon-dusted leaf of cowpea plants (*Vigna unguiculata* (L.) var California Blackeye) or benthamiana *Nicotiana benthamiana*, at the primary stage (two leaves). To demonstrate that the chimeric RNA3s encoded proteins, *in vitro* transcription and translation was performed using RNA transcription kit (mMessage Machine T7 kit, Ambion, Austin, TX, USA) and wheat germ extract translation kit (Promega, Madison, WI, USA).

Virus-like particle (VLP) production was accomplished by expression of wild-type, the salt-stable (SS) mutant, the subE (assembly deficient) mutant, and the five chimeric capsid proteins [4,5,10] in *Pichia pastoris* using expression plasmid pPicZA (Invitrogen, Carlsbad, CA, USA). Linearized plasmids were electroporated into *P. pastoris*, selected with zeocin (100 µg/mL), and grown in MGY medium (1.34% w/v yeast nitrogen base without amino acids (YNB), 1% v/v glycerol, 0.00004% w/v biotin), supplemented with methanol. Pseudovirions were isolated by freeze-thaw and lysis with acid-washed glass beads in an equal volume virus buffer (0.1 M sodium acetate/acetic acid, 1 mM sodium azide, 1 mM disodium EDTA, pH 4.8, 0.22 µm filtered). The mixture was vortexed vigorously four times for 5 min, with 2 min on ice in between. Cell debris and glass beads were separated by centrifugation at low speed. Chimeric VLPs in supernatant were precipitated by adding 10% (w/v) polyethylene glycol (PEG) MW 8000. The mixtures were centrifuged and the pellets were resuspended in virus buffer. VLPs were characterized by sucrose density centrifugation, SDS-PAGE, immunoblotting, and transmission electron microscopy.

### 2.2. Conjugation of Peptides to Carriers

S9 peptide (FDTGAFDPDWPA) with a C-terminal cysteine was conjugated to VLPs via the heterobifunctional cross-linking reagent sulfo-SMCC (Pierce Chemical, Rockford, IL, USA). To preclude aggregation between virus particles through disulfide linkages and prevent VLP cysteines from binding the maleimide on the cross-linking agent, VLPs were treated with tris (2-carboxyethyl) phosphine (TCEP) and 2-iodoacetamide. The peptides were TCEP-treated, and linked at a 50:1 coupling ratio. Coupling was confirmed by SDS-PAGE and ELISA. Integrity of conjugated VLPs was demonstrated on sucrose density gradients and by electron microscopy. The peptide was conjugated to maleimide-derivatized KLH (Pierce) at a mass ratio of 1:1. Conjugate was separated from peptide by centrifugal filtration on a 100 KD cutoff Microcon membrane (Millipore, Billerica, MA, USA). Antigen concentrations were determined by BCA Protein Assay (Pierce). Peptide antigen was quantified by ELISA with conjugate-coated microtiter wells and detected with the S9 mAb.

### 2.3. Immunization

Two separate immunizations were performed. In the first, female BALB/c mice 8–10 weeks old were immunized with antigens, either emulsified in a 1:1 ratio with complete Freund’s adjuvant (CFA, Difco, Detroit, MI, USA) for the primary immunization, and with incomplete Freund’s adjuvant (IFA) for booster injections (adjuvant group), or diluted with PBS for primary and booster immunizations (no adjuvant group). Each group was separated into five subgroups (four mice in each subgroup), each receiving a different antigen: CCMV, CCMV-S9, SubE-S9, CPMV-S9 and KLH-S9. Mice were immunized with following amount of antigens: CCMV-S9 20 µg, SubE-S9 20 µg, CPMV-S9 10 µg, KLH-S9 10 µg. The concentration of antigen was based upon ELISA results characterizing the amount of S9 peptide present. Mice were bled to collect sera before immunization (bleed 1). Primary immunization was given subcutaneously (sc) and boosters intraperitonealy (ip). Booster immunization followed the primary immunization by four weeks and one week after boosting, all mice were bled to collect sera (bleed 2) to measure antibody. A subset of mice were further boosted with 2.7 × 10^6^ heat-killed GBS. In the second experiment, 24 mice were separated into four groups receiving CCMV, CCMV-S9, CPMV-S9 and KLH-S9, all without adjuvant, using the same doses and timing. All animal protocols were approved by the IACUC at the Research Institute for Children. The mouse colony was AAALAC-approved.

### 2.4. ELISA

Antibody to peptide, CCMV, or GBS was measured by ELISA. Microtiter wells were coated with peptide or CCMV at 5 µg/mL, or with GBS immobilized as previously described [11]. Wells were blocked with PBS/1% bovine serum albumin. Sera were diluted to appropriate concentrations in PBS to measure different antibody responses: 1:200 to test the antibody response to GBS, 1:5000 to test the antibody response to S9 peptide, and 1:1000 for anti-CCMV. Sera were incubated 16 hours at 4 °C in antigen-coated microtiter wells. Wells were washed, and incubated with alkaline phosphatase-conjugated anti-mouse Ig class or subclass-specific antibodies (Zymed, South San Francisco, CA, USA). After 4–6 hours incubation the wells were washed and the colorimetric substrate *p*-nitrophenol in diethenaolamine buffer pH 9.8. Microtiter wells were read at A_405_ in an automated microplate reader (Biotek, Winooski, VT, USA).

### 2.5. Cytokine Assays

Mice from the second experiment with high anti-S9 and anti-GBS antibody titers were bled and immunized ip with the same antigen and dose as in the prime and boost. Immunized and age-matched naive mice were bled and sacrificed four days after the second booster immunization. Spleens and mesenteric lymph nodes were removed aseptically. The spleens and mesenteric nodes were pooled, single-cell suspensions prepared, and purified with Lymphocyte M separation medium (Cedarlane Laboratories Limited, Ontario, Canada). Cells were plated 100 µL per well (2 × 10^5^ cell per well) into sterile 96-well microplates (Costar, Corning Inc., Corning, NY, USA) and stimulated either with medium, CCMV, CCMV-S9, S9, KLH-S9, KLH, all at 20 µg/mL, or PHA-M (Sigma Chemical, St. Louis, MO, USA) at 10 µg/mL. Cultures were incubated for seven days at 37 °C in 5% CO_2_. The supernatants were collected at day 3, 5 and 7 and frozen. A sandwich ELISA was used to determine the amount of gamma interferon (IFN-γ), interleukin 4 (IL-4) and interleukin 10 (IL-10) produced by the cells during antigen activation. Antibody pairs, recombinant standards and ELISA reagent for the assay were purchased from eBioscience, Inc. Plates were coated with capture antibody. After blocking, the culture media, and manufacturer’s standards, were added in duplicate to the plates in a two-fold dilutions. We then added 100 µL/well of the diluted biotin-conjugated detection antibody, followed by avidin-peroxidase, and then 2,2-azinobis-3-ethylbenzthiazoline-sulfonic acid with 0.3% H_2_O_2_. The reaction was stopped with 0.7 M SDS in DMF and purified water, 1:1). The absorbance was measured at 450 nm. In each sample, the final concentration of IFN-γ, IL-4 or IL-10 was determined from the standard curves by calculating the mean concentration from two dilutions.

## 3. Results and Discussion

In an effort to reproduce the repeating carbohydrate structure of a microbial polysaccharide with a peptide mimetic and to enhance immunogenicity, we attempted to express the S9 peptide as a chimeric capsid protein on CCMV, which contains 180 copies per virion. Our first attempt was to produce infectious virus, expressing the S9 mimetic peptide in a surface-exposed loop of the capsid protein. RNA3 encodes the capsid protein, and five different plasmid constructs were produced, expressing the peptide in each of the surface-exposed loops. Although ample quantities of infectious virus were made with the wild-type, none was observed with any of the chimeric constructs. Because capsid function can play an important role in virus infectivity, we next attempted to express the chimeras as VLPs, expressed in the yeast *Pichia pastoris*. Wild-type sequences produced a large quantity of VLPs; however, none of the chimeras produced VLPs, although all made large quantities of the capsid protein. From these results we conclude that the introduction of extraneous peptide sequences within any of the surface-exposed loops of the CCMV capsid protein interferes with capsid assembly. Chimeric capsid proteins of other icosahedral viruses have been utilized to express foreign peptides, including poliovirus [12], human rhinovirus, [13], and the closely related plant virus CPMV [6], although in the latter case insertion of foreign sequences can have an effect on infectivity, host range, and biochemical phenotype of the resulting virions [14]. Attempts to insert other sequences into CCMV have generally been ineffective, although the insertion of the cell-targeting peptide 11 into position 129 did result in a viable CCMV chimera, albeit with greatly reduced infectivity [15].

**Figure 1:**
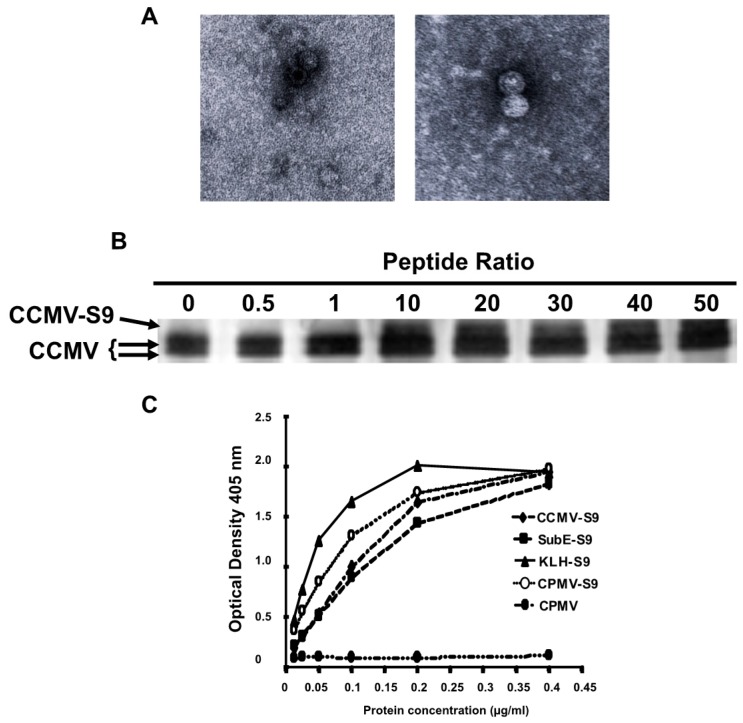
Production of VLPs and chemically conjugated VLPs. (**A**) Transmission electron micrographs of VLPs purified on a density gradient; (**B**) Coupling of S9 peptide to VLPs. VLPs of ssCCMV were incubated with cross-linking reagent at a 1:1 ratio of cross linker to capsid subunit. Different concentrations of the peptide were then added. After 1.5 hours, the reaction was stopped and samples loaded onto non-reducing 12% SDS-PAGE gels and visualized by silver stain. Free and peptide-conjugated capsid protein was observed; (**C**) Measurement of S9-peptide on different immunogens. The protein concentration of the different immunogens was determined by the BCA method. Different concentrations were coated onto ELISA wells. Peptide was detected with the S9 mAb, then alkaline phosphatase-conjugated anti-mouse IgM.

To produce VLPs that express the S9 peptide sequences, we constructed an S9 peptide with a C-terminal cysteine, and coupled the peptide to *Pichia*-expressed VLPs using the heterobifunctional cross-linking reagent SMCC. For this purpose we used the salt-stable mutant of CCMV, which will not disassemble under the conditions of the chemical conjugation [4,5,10]. Figure 1A shows transmission electron micrographs of two different VLP preparations demonstrating the presence of capsids. Figure 1B demonstrates that approximately 20%–30% of the capsid proteins are derivatized by the S9 peptide, representing 40–50 copies of the peptide per virus particle. A full-length capsid is 20.3 KD, whereas an S9-conjugated capsid is 21.8 KD.

Five different immunogens were made: (1) salt-stable CCMV VLPs, (2) salt-stable CCMV VLPs conjugated to the S9 peptide as demonstrated above, (3) SupE CCMV monomer capsid proteins conjugated to the S9 peptide, (4) CPMV VLPs conjugated to the S9 peptide, and (5) KLH conjugated to the S9 peptide via a maleimide cross-linker. To determine the relative amounts of peptide attached to each carrier, we performed an ELISA in which the S9 peptide was detected with the S9 monoclonal anti-GBS antibody (Figure 1C). On the basis of these results, the amount of each immunogen was normalized so that each animal received the same amount of the S9 peptide. To achieve this, the following doses of each immunogen were used: CCMV-S9 20 µg, SubE-S9 20 µg, CPMV-S9 10 µg, KLH-S9 10 µg.

**Figure 2:**
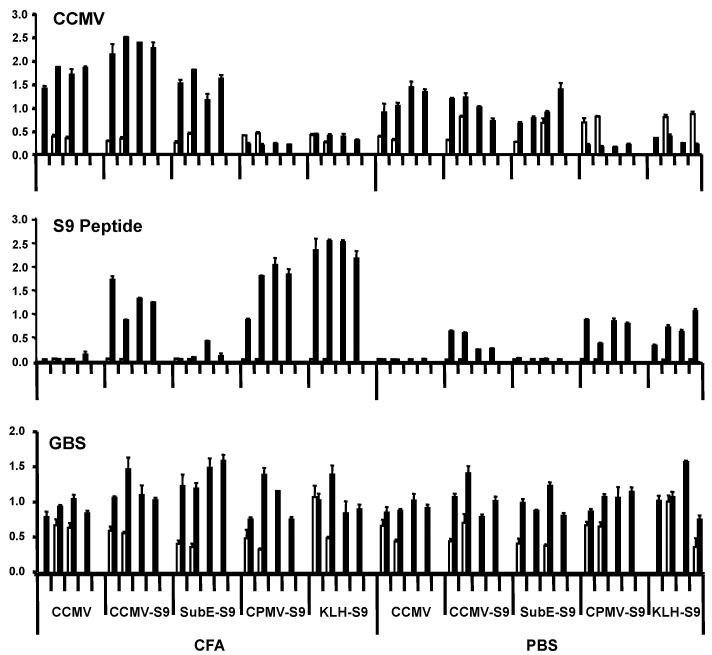
Production of antibody to CCMV, S9 peptide, and GBS by mice immunized with different immunogens. Groups of four mice were immunized with ssCCMV VLPs, ssCCMV VLPs conjugated to S9 peptide, subE (monomeric capsid) conjugated to S9 peptide, CPMV VLPs conjugated to S9 peptide, and KLH conjugated to S9 peptide. Mice were immunized either with Freund’s adjuvant, or in the absence of adjuvant. The results of individual mice are shown. Prebleeds are in open bars, sera obtained following immunization in solid bars. ELISA plates were coated with the indicated antigens. Sera were tested at 1:1000 dilution for CCMV, 1:5000 for S9 peptide, and 1:200 for GBS. Binding was detected with alkaline phosphatase conjugated anti-mouse IgG (H + L). Antibodies of all isotypes are detected because of the L chain specificity of the conjugated antiserum.

In the first experiment, groups of four mice were immunized with the five antigens, either with Freund’s adjuvant (complete for the primary immunization, incomplete for the booster immunization) or in saline. Mice received primary immunization subcutaneously, and four weeks later, one booster immunization intraperitoneally. They were bled prior to the primary immunization and one week following the booster. Antibody responses were measured against CCMV, S9 peptide, and GBS (Figure 2). Responses against the S9 peptide and CCMV were measured at a serum dilution of 1:5000 and 1:1000, respectively. We have found that the level of antibody at this dilution (as indicated by ELISA OD) correlates well with the titer, and have therefore presented our results in this manner. All mice immunized with CCMV-containing antigens produced anti-CCMV antibody, with the adjuvant group having somewhat higher levels of antibody. Immunization with CPMV did not elicit cross-reactive anti-CCMV antibody. Antibody against the S9 peptide was elicited by all S9-containing antigens, except for the subE CCMV (monomer capsid protein)-S9 conjugate. Even more than with the anti-CCMV response, the adjuvant provoked a greater anti-S9 antibody response than in its absence. The failure of the subE CCMV-S9 to elicit an anti-S9 response supports the recent finding of the critical function of multimerization in eliciting antibody to epitopes expressed on virus-like particles [16]. The results with anti-GBS antibody were somewhat more difficult to assess. All experimental groups, including controls receiving no S9 peptide, had higher levels of anti-GBS antibody following the immunizations than in the prebleed. The results suggest that mice developed anti-GBS antibody while living in our animal colony, regardless of immunization. This has been confirmed by placing mice within the colony and observing the development of anti-GBS antibody with time. This occurs regardless of the strain of mouse, and in two different mouse colonies, both of which are maintained as specific pathogen–free colonies (in Bozeman, MT, and New Orleans, LA, USA). Repeated attempts to culture GBS from the mice have yielded negative results. These results suggest that mice are exposed to a cross-reactive antigen, either as part of the normal flora or in the environment (e.g., feed or bedding). The “natural” development of anti-GBS antibody confounds the interpretation of immunization results.

**Figure 3:**
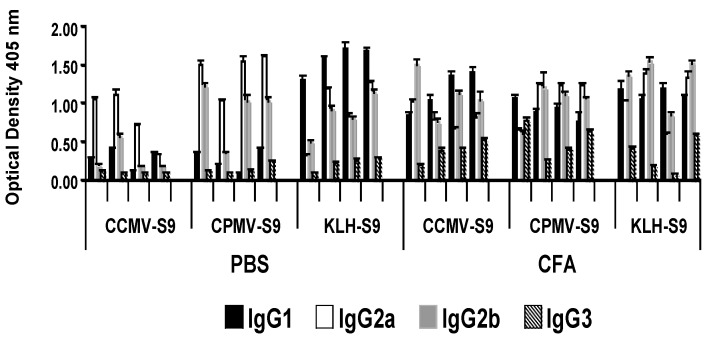
IgG subclasses of anti-S9 antibody response. Post-immune sera of individual mice immunized with the indicated antigens were tested by ELISA in wells coated with the S9 peptide. Antibodies of the different IgG subclasses were detected with alkaline phosphatase-conjugated subclass-specific antibodies.

In the groups of mice producing the highest levels of anti-S9 peptide antibody, we studied the IgG subclass of the anti-peptide antibody response (Figure 3). Mice immunized with antigen emulsified in Freund’s adjuvant produced equivalent levels of IgG1, IgG2a, and IgG2b antibody. However, there were marked subclass differences in mice immunized with antigen in saline. Mice immunized with VLPs, CCMV or CPMV conjugated to the peptide produced a dominant IgG2a response, whereas mice that received KLH-S9 produced a response dominated by IgG1.

To determine whether immunization with S9 peptide primed for a secondary immune response to GBS, mice immunized with CCMV, CCMV-S9, and KLH-S9 received an injection of 2.7 × 10^6^ heat-killed GBS. The mice were bled prior to injection, as well as three and seven days post-injection. We tested for IgG and IgM responses to GBS and S9 peptide. The results demonstrated that by day 7 all mice, including those primed with the control immunogen, unconjugated CCMV, produced high levels of IgM antibodies to both GBS and the S9 peptide. These results confirm that the mice are capable of a response to GBS, and also demonstrate that anti-GBS antibodies cross-react with the S9 peptide, the original finding that initiated this series of studies [1]. Levels of IgG anti-GBS rose only modestly, again in all groups.

**Figure 4:**
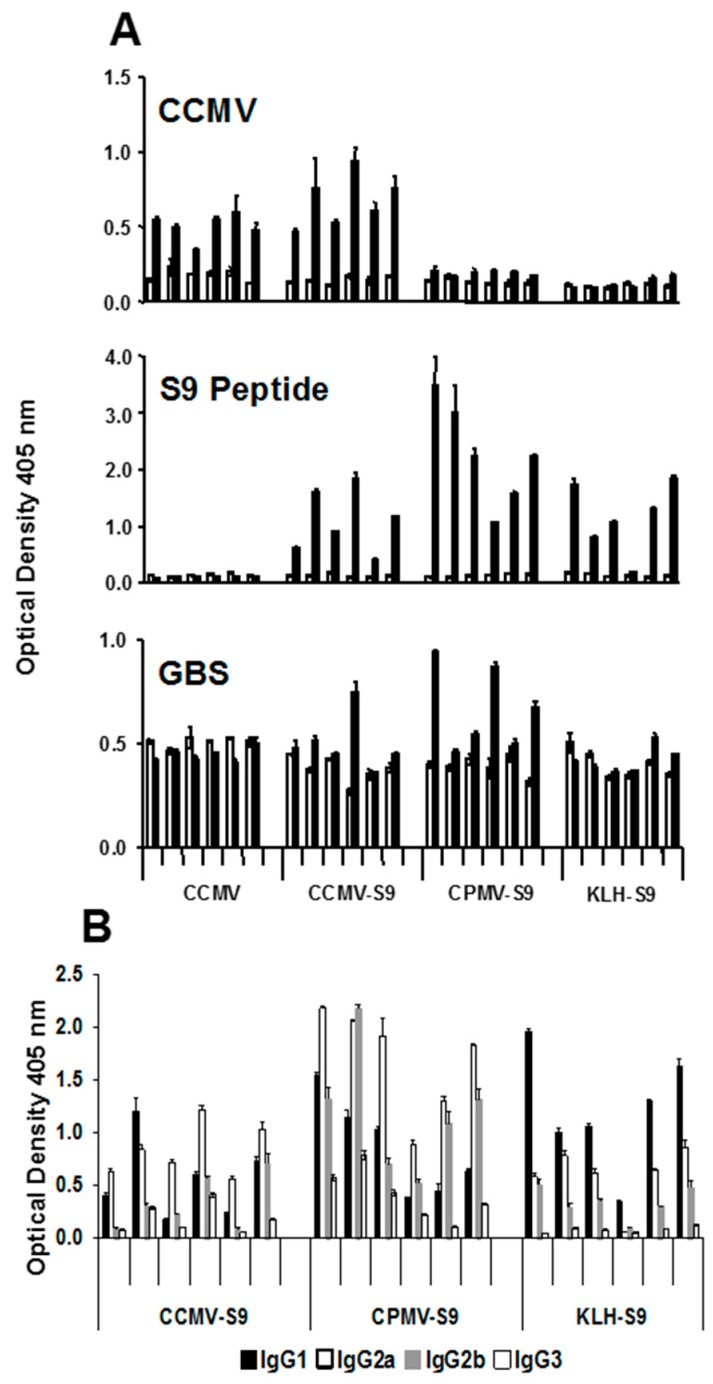
Antibody responses in a second experiment. Groups of six mice were immunized with CCMV, CCMV-S9, CPMV-S9, and KLH-S9 with no adjuvant. In panel A, prebleed (open bar) and post-immune serum (filled bar) were tested for binding to CCMV, S9 peptide, and GBS and detected with alkaline phosphatase–conjugated anti-mouse IgG (H + L). In panel B, the sera are tested for IgG subclass of antibody to the S9 peptide.

To confirm the effect of the different immunogens on the antibody responses, a second experiment was performed in which mice were immunized with CCMV (control), CCMV-S9, CPMV-S9, and KLH-S9 in saline. The antibody response to CCMV, S9 peptide, and GBS was measured, as were the IgG subclasses of the anti-S9 peptide response (Figure 4). The results basically duplicate those responses observed in the first experiment. An anti-CCMV antibody response was observed in mice immunized with CCMV and CCMV-S9, but not those receiving CPMV-S9 nor KLH-S9. An anti-peptide response was seen in all but the control mice, with the CPMV-S9-immunized mice producing the best response. In this experiment, the control-immunized mice had the same, rather high level of anti-GBS antibody both before and after the immunizations. In contrast, three of five mice immunized with CPMV-S9 had increased levels of anti-GBS antibody. Only one of five mice immunized with CCMV-S9 and none immunized with KLH-S9 had similar elevations. The IgG subclass distribution of anti-peptide antibody (Figure 4B) demonstrated the VLP-associated immunogen gave more IgG2a/2b antibody (except in one mouse), whereas the dominant subclass in the KLH-S9-immunized group was IgG1.

**Figure 5:**
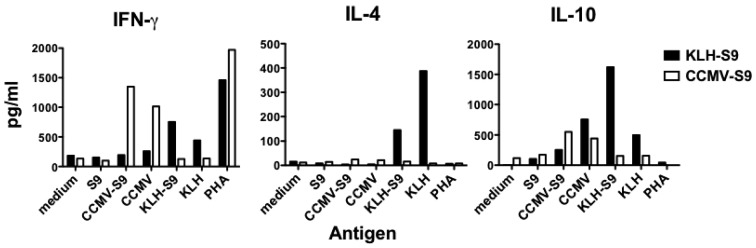
Cytokine production by lymphocytes from VLP- or carrier-immunized mice. Mice immunized with CCMV-S9 or KLH-S9 were boosted and, four days later, sacrificed. Lymphocytes from mesenteric lymph nodes and spleens were pooled from mice in each group and splenocytes isolated. The cells were incubated with the indicated antigens and cytokines in the cell supernatants measured on day 3 (IFN-γ) or day 5 (IL-4 and IL-10) using cytokine capture ELISAs.

Because the results of the IgG subclass analysis suggested that the antibody response to the S9 peptide was biased either towards Th1 (VLP-associated antigen) or Th2 (antigen conjugated to KLH), we examined the cytokines released by splenocyte cells from mice immunized with CCMV-S9, or KLH-S9 from experiment 2. Four days following a booster immunization, splenocytes were purified and incubated with S9 peptide, CCMV, CCMV-S9, KLH, KLH-S9, the mitogen phytohemagglutinin (PHA) or medium alone. The Th1/Th2 cytokines IFN-γ, IL-4, and IL-10 were measured (Figure 5). The results demonstrate that for both IFN-γ and IL-10, the carriers elicited a cytokine response, for example CCMV-S9-immune mice produced IFN-γ and IL-10 in response to CCMV and CCMV-S9, but not to KLH or KLH-S9. CCMV elicited a higher level of IFN-γ than did KLH, whereas KLH elicited more IL-10 than CCMV. However, IL-4 was elicited only by KLH and KLH-S9 in the KLH-S9-immune animals. As expected, S9 peptide alone failed to elicit any response. Naïve mice did not produce cytokines in response to any antigen. These data suggest that the stimulation of IL-4 production by the carrier protein KLH is responsible for the generation of a Th2 response. These results support the observations that multimeric VLP vaccines induce a Th1-biased immune response [17,18,19], whereas epitopes expressed on carriers are more likely to elicit Th2 responses [17]. Because the pseudoviruses used as carriers package RNA during the assembly process [5], we speculate that this effect may result from the presence of nucleic acid in the immunogen, acting through stimulation of innate immune receptors. However, we have not tested this hypothesis experimentally.

## 4. Conclusions

The results presented in this manuscript demonstrate the following: (1) Attempts to create chimeric CCMV by inserting foreign peptide sequences into any of the surface-exposed loops of the capsid protein result in failure of virus/VLP assembly; (2) Immunization with peptide conjugated to yeast-expressed VLPs results in a Th1 response directed against the peptide, whereas if the peptide is conjugated to the protein carrier, a Th2 response develops. This polarity appears to result from the carrier-driven secretion of IL-4, and is not observed when adjuvant is used; (3) The S9 peptide is an antigenic mimic, in that immunization of mice with GBS results in the production of S9 peptide–cross-reactive antibody [1]. However, it is less effective as a mimetic immunogen, inducing marginal anti-GBS responses and no immunological memory for GBS.

## Abbreviations

The following abbreviations are used in this manuscript:

CCMV: cowpea chlorotic mottle virus
CPMV: cowpea mosaic virus
ELISA: enzyme-linked immunosorbent assay
GBS: group B Streptococcus, or *Streptococcus agalactiae*
ip: intraperitoneal
KLH: keyhole limpet hemocyanin
PEG: polyethylene glycol
S9: originally an anti-GBS monoclonal antibody, but now also the peptide bound by that antibody
sc: subcutaneous
VLP: virus like particle
